# Functional and Epigenomic Consequences of *DNMT1* Variants in Inherited Neurological Disorders

**DOI:** 10.3390/ijms27031232

**Published:** 2026-01-26

**Authors:** Jun-Hui Yuan, Yujiro Higuchi, Masahiro Ando, Akiko Yoshimura, Satoshi Nozuma, Yusuke Sakiyama, Takashi Kanda, Masahiro Nomoto, Takeshi Nakamura, Yasuyuki Nobuhara, Hiroshi Takashima

**Affiliations:** 1 Department of Neurology and Geriatrics, Kagoshima University Graduate School of Medical and Dental Sciences, Kagoshima 890-8520, Japan; 2 Department of Neurology and Clinical Neuroscience, Yamaguchi University Graduate School of Medicine, Yamaguchi 755-8505, Japan; 3 Department of Neurology and Clinical Pharmacology, Ehime University Graduate School of Medicine, Toon 791-0295, Japan; 4 Department of Neurology, Osaka Red Cross Hospital, Osaka 543-8555, Japan; 5 Department of Neurology, National Hospital Organization Minami-kyushu Hospital, Kagoshima 899-5293, Japan

**Keywords:** *DNMT1*, sensory neuropathy, spinocerebellar ataxia, methylation, 5mC

## Abstract

*DNMT1* variants are linked to complex neurodegenerative syndromes, yet their variant-specific functional and epigenomic consequences remain poorly defined. *DNMT1* variants were identified in eight patients using gene-panel or whole-exome sequencing. Functional effects were assessed by site-directed mutagenesis and transient expression in HEK293T cells. Genome-wide methylation profiling of peripheral blood leukocyte DNA was performed using Nanopore sequencing, enabling direct quantification of 5-methylcytosine (5mC). CpG island-level differential methylation and gene set enrichment analysis (GSEA) were conducted. Variants in the replication foci targeting sequence (RFTS) domain (p.Y511H, p.Y540C, p.H569R) exhibited reduced DNMT1 protein expression, decreased enzymatic activity, and cytosolic aggregation. Variants in the C-terminal catalytic domain (p.A1334V and p.P1546S) showed reduced protein expression with relatively mild enzymatic impairment. Patients carrying the p.Y511H variant demonstrated a significant reduction in global 5mC levels compared with controls. Principal component analysis revealed distinct methylomic profiles separating most patients from controls, with marked intra- and inter-familial heterogeneity. CpG island-level analysis identified a single significantly hypomethylated region in p.Y511H carriers, and GSEA revealed differential enrichment of multiple Gene Ontology biological pathways. This study defines domain-dependent functional effects of *DNMT1* variants and provides the first nanopore-based methylome analysis, revealing variant-specific and heterogeneous epigenomic alterations.

## 1. Introduction

DNMT1 is a key enzyme that maintains DNA methylation patterns during cell division and development. It recognizes hemimethylated CpG dinucleotides and transfers methyl groups to the corresponding cytosines on the daughter strand, generating 5-methylcytosine (5mC) [1]. CpG methylation is pervasive across mammalian genomes and fulfills distinct context-dependent roles: methylation within CpG islands regulates gene expression, genomic imprinting, and X-chromosome inactivation [2,3], whereas methylation within gene bodies correlates with transcriptional activity [4].

Pathogenic variants in *DNMT1* cause a spectrum of neurodegenerative disorders, including hereditary sensory and autonomic neuropathy type IE (HSAN IE; OMIM: 614116) and autosomal dominant cerebellar ataxia with deafness and narcolepsy (OMIM: 604121), reflecting complex multisystem phenotypes [5,6,7]. To date, more than 30 disease-associated *DNMT1* variants, predominantly missense substitutions, have been reported (Human Gene Mutation Database, accessed January 2025), most of which cluster within the replication foci targeting sequence (RFTS) domain, a region essential for nuclear localization and enzymatic regulation [8]. Genome-wide methylation studies of RFTS variants, based on bisulfite-treated DNA analyzed by microarray or short-read whole-genome bisulfite sequencing (WGBS), have consistently demonstrated global hypomethylation [6,9,10].

Recent advances in long-read sequencing, such as Oxford Nanopore technology, enable direct analysis of native genomic DNA, providing 5mC profiles highly concordant with WGBS [11,12,13] and permitting simultaneous detection of its downstream derivative, 5-hydroxymethylcytosine (5hmC). In this study, we combined functional analyses in HEK293T cells with nanopore-based genome-wide methylation profiling to investigate both known and novel *DNMT1* variants. This approach provides a framework for evaluating variants of uncertain significance and offers new insights into the genotype–phenotype relationships underlying *DNMT1*-related disorders.

## 2. Results

### 2.1. Clinical Summary and Genetic Findings

Patient 1: A 37-year-old man presented with the onset at around age 16 of severe distal sensory loss, resulting in recurrent osteomyelitis and toe amputations, accompanied by progressive bilateral sensorineural hearing loss. Examination revealed absent pain and touch sensation in the distal lower limbs, positive Romberg sign, and absent deep tendon reflexes. Mild intellectual impairment was noted. Brain MRI demonstrated mild diffuse cerebral and cerebellar atrophy. Electrophysiology showed absent sensory nerve action potentials (SNAP) in the median, ulnar, and sural nerves [14]. Gene-panel sequencing identified a heterozygous *DNMT1* variant, NM_001130823.3:c.1706A > 0020G (p.H569R), in this patient.

Patient 2: A 50-year-old man presented with congenital bilateral hearing loss and cataracts. Recurrent ileus began in adolescence, and distal sensory disturbances emerged in his late 20s, followed by orthostatic hypotension. Examination revealed distal sensory loss, hyporeflexia, and borderline cognitive impairment. Electrophysiology demonstrated sensory-dominant axonal polyneuropathy, and brain MRI showed mild diffuse cerebral atrophy [15]. A *DNMT1* missense variant (c.4001C > T, p.A1334V) was detected by gene-panel sequencing. This variant was absent in the unaffected mother and sister but was present in population databases (rs766051225; gnomAD: East Asian = 0; South Asian = 11/30,614; total = 14/27,9570; jMorp: 64/108,604).

Patient 3: A 42-year-old woman developed progressive hearing loss and gait disturbance beginning at age 20. In her 30s, she experienced amenorrhea, and hypogonadotropic hypogonadism was confirmed. Neurological examination revealed cognitive impairment (IQ 68), bilateral sensorineural hearing loss, distal paresthesia, hyporeflexia in the lower limbs, and cerebellar ataxia. Brain MRI demonstrated mild cerebellar atrophy. Nerve conduction studies showed absent SNAP in all tested nerves. Gene-panel sequencing identified a novel heterozygous *DNMT1* variant (c.1619A > G, p.Y540C).

Patient 4: A 35-year-old woman with a positive family history (aunt, cousin, and deceased father) presented with progressive ataxia beginning at age 35, characterized by gait instability, nystagmus, and impaired fine motor coordination. She had cataracts and glaucoma. Pain sensation was intact, but vibration sense was reduced, and sural SNAPs were decreased. Hearing, motor, and autonomic functions were preserved. Brain MRI revealed cerebral atrophy, while cognitive function remained normal. This patient was clinically suspected of having spinocerebellar ataxia (SCA), and whole-exome sequencing (WES) identified a novel *DNMT1* variant, c.4636C > T (p.P1546S), which showed complete segregation with disease among all affected family members.

Patient 5 (II-2, index case): A 52-year-old man with a positive family history presented in his 40s with hearing loss, gait disturbance, and dysarthria. Examination revealed mild cognitive impairment (Mini-Mental State Examination, MMSE 25), bilateral sensorineural hearing loss, explosive speech, distal lower-limb paresthesia with reduced sensation, absent lower-limb reflexes, and limb and truncal ataxia. Brain MRI showed moderate diffuse cerebral and cerebellar atrophy. Nerve conduction study (NCS) indicated pure sensory axonal neuropathy. DNA samples were collected from multiple affected cousins, and their clinical features have been previously reported [16]. WES revealed a known *DNMT1* variant, c.1531T > C (p.Y511H), which was shared by the other three affected family members.

**Figure 1:**
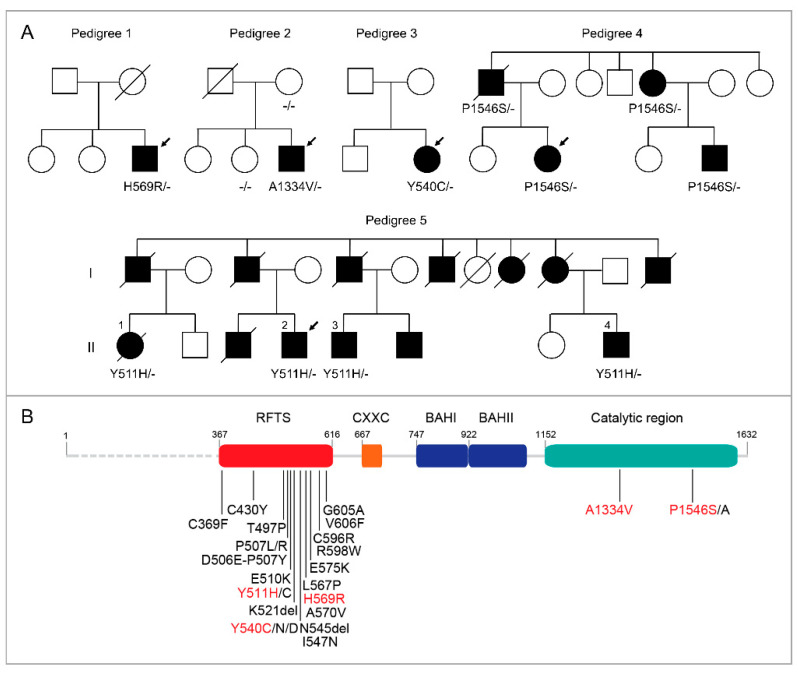
Pedigree analysis and distribution of *DNMT1* variants. (**A**): Segregation of *DNMT1* variants in five pedigrees. Index cases are indicated by arrows. (**B**): Schematic representation of the DNMT1 protein structure (PDB ID: 7XI9), showing previously reported variants (black) and variants identified in this study (red). Key functional domains are labeled: RFTS, replication foci targeting sequence; CXXC, CXXC DNA binding domain; BAH, bromo-adjacent homology domain.

These clinical and genetic features are summarized in Table 1 and Figure 1A.

### 2.2. Distribution of DNMT1 Variants

Among the identified variants, p.Y511H, p.Y540C, and p.H569R are located within the RFTS domain of DNMT1 (amino acids 351~600), whereas p.A1334V and p.P1546S reside in the C-terminal catalytic region. (Figure 1B)

### 2.3. Conserved DNMT1 mRNA Expression in HEK293T Cells Expressing DNMT1 Variants

**Figure 2:**
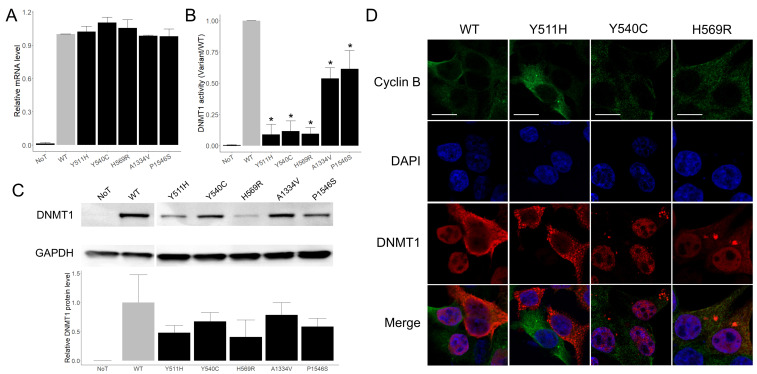
*DNMT1* mRNA expression, protein levels, and enzymatic activity in transiently transfected HEK293T cells. (**A**): RT-qPCR analysis showing no significant differences in *DNMT1* mRNA expression between WT and variant constructs. (**B**): DNMT1 enzymatic activity assay demonstrating significant reduction in activity for all RFTS-domain variants, with a moderate but significant decrease also observed for p.A1334V and p.P1546S. (**C**): Western blot analysis of whole-cell lysates indicating a trend toward reduced DNMT1 protein levels in cells expressing *DNMT1* variants compared with WT. Statistical analyses were performed using one-way ANOVA with independent experiments (*n* = 3); *p* < 0.01 is indicated by an asterisk (*). NoT: non-transfected. (**D**): Immunofluorescence images showing cytosolic DNMT1 aggregates in cells expressing RFTS-domain variants. Scale bar = 10 µM.

Wild-type (WT) *DNMT1* cDNA (pcDNA3.1-*DNMT1*-His) and five *DNMT1* variant constructs were transiently transfected into HEK293T cells. Total RNA was isolated and subjected to reverse transcription followed by RT-qPCR analysis. No significant differences in *DNMT1* mRNA expression levels were observed between cells expressing WT *DNMT1* and those expressing variant constructs (one-way ANOVA). (Figure 2A)

### 2.4. Reduced DNMT1 Enzymatic Activity and Protein Expression

Enzymatic activity assays performed using equal amounts of purified His-tagged DNMT1 protein (100 ng) revealed a significant reduction in DNMT1 activity for all three RFTS-domain variants, as well as a moderate but significant decrease for the p.A1334V and p.P1546S (one-way ANOVA followed by Dunnett’s multiple comparisons test; all *p* < 0.01) (Figure 2B).

Western blot analysis of whole-cell lysates showed a trend toward reduced DNMT1 protein levels across all variants, with the largest decreases observed for p.Y511H and p.H569R relative to WT DNMT1. However, these differences did not reach statistical significance (one-way ANOVA) (Figure 2C).

### 2.5. Cytosolic Aggregation of DNMT1 in RFTS-Domain Variants

Previous studies have shown that *DNMT1* variants affecting the RFTS domain can form cytosolic aggregates in a cell-cycle-dependent manner, particularly after S phase [5,6,17]. To determine whether similar aggregation occurs in our system, we examined DNMT1 localization by immunofluorescence in transiently transfected HEK293T cells. Cell-cycle status was approximated using cyclin B expression and subcellular localization as indicators of the G2/M phase. No endogenous DNMT1 signal was detected in non-transfected (NoT) HEK293T cells. In contrast, cells expressing RFTS-domain variants (p.Y511H, p.Y540C, and p.H569R) displayed cytosolic DNMT1 aggregates. These aggregates were observed mainly in cells lacking cyclin B expression, suggesting that aggregation was not restricted to the G2/M phase. In comparison, cells expressing C-terminal variants (p.A1334V and p.P1546S) showed a diffuse DNMT1 distribution and no evidence of cytosolic aggregation under the same conditions. (Figure 2D)

Because HEK293T cells proliferate asynchronously, cycling and cyclin B alone do not allow precise delineation of post-S phase cells; quantitative assessment of aggregation frequency was not performed. Thus, these observations qualitatively support an association between cytosolic aggregation and RFTS-domain *DNMT1* variants.

### 2.6. Global DNA Methylation Levels in Peripheral Blood Leukocytes

**Figure 3:**
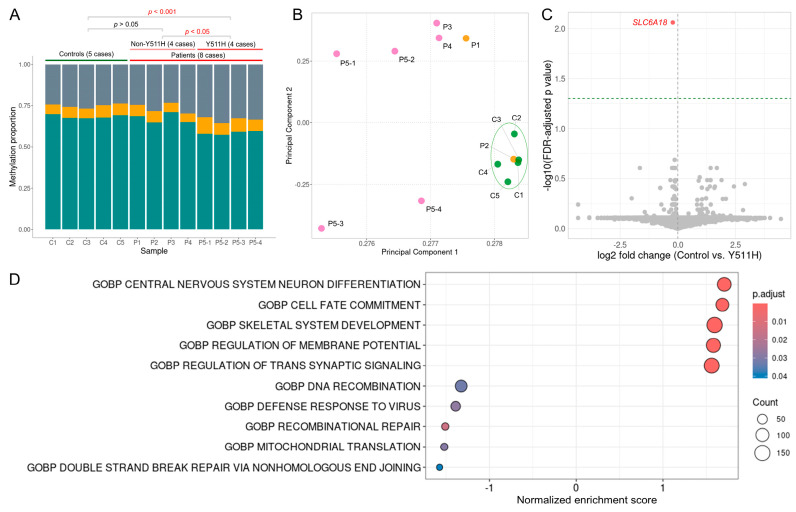
Global and CpG island-based methylation analysis using nanopore sequencing. (**A**): Global 5mC levels are significantly reduced in patients carrying the p.Y511H variant compared with controls (Welch’s one-way ANOVA with Games–Howell post hoc test; *p* < 0.001) and with non-p.Y511H patients (*p* < 0.05). No significant difference is observed between the control and non-p.Y511H groups. Cyan: 5mC; orange: 5hmC; gray: unmethylated. (**B**): Principal Component Analysis (PCA) of CpG island methylation profiles shows clustering of five controls and P2 (p.A1334V), distinct from other patient samples. Green dots: controls; pink dots: patients with cerebellar ataxia; orange dots: patients with sensory neuropathy without cerebellar involvement. (**C**): Volcano plot of CpG island-level differential methylation analysis between controls and p.Y511H carriers, highlighting a single significantly hypomethylated CpG island within the *SLC6A18* gene in p.Y511H patients (Benjamini–Hochberg FDR-adjusted *p* < 0.01). (**D**): Gene set enrichment analysis reveals differential enrichment of Gene Ontology biological process terms between controls and the p.Y511H group.

To assess genome-wide DNA methylation, nanopore long-read sequencing was performed on peripheral blood leukocyte DNA from eight patients harboring *DNMT1* variants and five age-matched controls. Sequencing yielded an average of 19.88 ± 3.13 Gb of high-quality data per sample over a 72 h run. Patients carrying the p.Y511H variant exhibited a marked reduction in global 5mC levels compared with controls (0.585 ± 0.01 vs. 0.683 ± 0.01; Welch’s one-way ANOVA with Games–Howell post hoc test; *p* < 0.001). In contrast, global 5mC levels in patients with non-p.Y511H *DNMT1* variants (0.673 ± 0.03) were not significantly different from controls (*p* > 0.05). Global 5mC levels were also significantly lower in the p.Y511H group compared with the non-p.Y511H group (*p* < 0.05). (Figure 3A)

Notably, global 5mC levels among carriers of other RFTS-domain variants were heterogeneous. The p.H569R carrier (P1) showed levels comparable to controls (0.685), whereas the p.Y540C carrier (P3) exhibited higher levels than controls (0.711), indicating variant-specific methylation effects. In contrast, global 5hmC levels showed substantial inter-individual variability, with no statistically significant differences among controls, p.Y511H, and non-p.Y511H groups (Welch’s one-way ANOVA) (Table 2).

### 2.7. Genome-Wide Methylation Profile in CpG Islands

CpG islands were annotated using the UCSC Genome Browser. CpG islands on chromosomes X and Y, as well as those with missing data in any sample, were excluded to minimize sex-related and technical variability. A total of 25,611 CpG islands were retained for analysis, with a mean sequencing depth of 6.57 ± 1.05. Mean CpG island methylation was calculated per sample and compared across groups. The non-p.Y511H group showed the highest mean CpG island methylation (27.44%), followed by controls (25.70%) and the p.Y511H group (25.26%). However, these differences were not statistically significant (Welch’s one-way ANOVA).

Unsupervised Principal Component Analysis (PCA) based on these CpG island methylation levels was performed to visualize methylomic heterogeneity among samples. All five control samples clustered tightly together, along with P2 carrying the p.A1334V variant, indicating broadly similar methylation profiles. In contrast, the remaining *DNMT1* variant carriers were clearly separated from controls and from each other, highlighting deviation from typical methylation patterns and substantial inter-individual heterogeneity among *DNMT1*-related patients, including within the same pedigree (Figure 3B).

### 2.8. Differential Methylation Analysis of CpG Islands

To identify CpG island-level differentially methylated regions (DMRs), methylation profiles from four patients carrying the p.Y511H variant (P5-1 to P5-4) were compared with those from five controls. Group comparisons were performed at the CpG island level using Welch’s two-sided t-tests, with multiple testing controlled by the Benjamini–Hochberg false discovery rate (FDR). This analysis identified a single CpG island (chr5:1,226,861-1,227,190) within the *SLC6A18* gene that was significantly hypomethylated in the p.Y511H group (FDR-adjusted *p* value < 0.01) (Figure 3C). A complete list of CpG islands and DMR statistics is provided in Supplementary Table S1.

### 2.9. Gene Set Enrichment Analysis (GSEA)

To facilitate functional interpretation of CpG island-level methylation changes, CpG islands were assigned to genes based on UCSC HGNC annotations. GSEA was then performed using fold changes in mean gene-level CpG island methylation between control and p.Y511H groups, with statistical significance assessed using Benjamini–Hochberg FDR correction.

Using the clusterProfiler GSEA implementation, we identified significant enrichment of multiple Gene Ontology (GO) biological process terms. Pathways enriched in the p.Y511H group included neuropeptide signaling pathway, cell fate specification, and positive regulation of neuron differentiation, whereas pathways enriched in controls included double-strand break repair via non-homologous end joining, mitochondrial translation, and recombinational repair (FDR < 0.05). The top five pathways ranked by normalized enrichment score (NES) for each group are shown in Figure 3D, with full GSEA results provided in Supplementary Table S2.

## 3. Discussion

In this study, we performed an integrated functional and epigenomic characterization of multiple patient-derived *DNMT1* variants associated with complex neurodegenerative phenotypes. By combining site-directed mutagenesis and cellular assays in HEK293T cells with genome-wide DNA methylation profiling using nanopore long-read sequencing, we delineated variant-specific molecular consequences and provided a structured framework for evaluating *DNMT1* variants across functional domains.

DNMT1 comprises several functional domains, including the RFTS, CXXC, two bromo-adjacent homology (BAH), and the C-terminal catalytic domain [18]. The RFTS domain plays a critical role in DNMT1 autoinhibition and in recruitment to replication foci through interaction with UHRF1, thereby coordinating methylation maintenance during DNA synthesis [8,19]. Consistent with this role, all RFTS-domain variants examined in this study (p.Y511H, p.Y540C, p.H569R) exhibited markedly reduced DNMT1 protein levels and enzymatic activity, despite unaltered mRNA expression. These findings indicate post-transcriptional effects such as impaired protein stability or misfolding. In line with previous reports, mutant DNMT1 proteins formed cytosolic aggregates in transfected cells, supporting a misfolding- and aggregation-driven mechanism [5,6,17]. Aggregate morphology varied among variants, suggesting heterogeneity in protein destabilization. Using Cyclin B1 as a cell-cycle indicator, we observed that aggregate formation was not restricted to the G2/M phase, indicating that aggregation is not strictly coupled to mitosis but may arise from impaired nuclear import or intrinsic instability during interphase.

Reduced enzymatic activity was consistently observed across all RFTS-domain variants, supporting the concept that substitutions in this domain disrupt intramolecular regulation of DNMT1. Structural studies have shown that perturbation of interactions between the RFTS and catalytic lobes can substantially alter enzyme activity [20], and our results are broadly consistent with previous functional analyses of RFTS-domain variants [6,21]. At the same time, reports of increased activity for certain substitutions, such as p.A554V [22], highlight the complexity of DNMT1 regulation and emphasize the need for variant-specific interpretation.

Clinically, patients harboring RFTS-domain variants presented with a characteristic but variable spectrum of neurological features, including progressive sensory neuropathy, hearing loss, cognitive impairments, and cerebellar ataxia (notably in P3 and P5). Electrophysiological findings were consistent with sensory-dominant axonal neuropathy. The novel p.Y540C variant (P3) was associated with a phenotype similar to that reported for another substitution at the same residue, p.Y540N [23], supporting the functional importance of this position.

Despite shared molecular features, clinical severity varied across patients. For example, the p.H569R carrier (P1) showed particularly severe sensory complications, including recurrent osteomyelitis, a feature previously reported in *DNMT1*-related neuropathy [5,6,17]. Such variability likely reflects variant-specific effects within the RFTS domain, leading to differential disruption of DNMT1 stability, chromatin binding, or regulatory interactions, and underscores the absence of a simple genotype–phenotype relationship.

Beyond the RFTS domain, we evaluated variants affecting the catalytic region of DNMT1. The p.A1334V variant, originally reported by our group [15], showed a moderate reduction in enzymatic activity but exhibited a methylation profile largely overlapping with controls. Together with its presence at low frequency in population databases, these findings suggest that p.A1334V may exert a mild functional effect that is insufficient, on its own, to consistently cause disease.

We also identified a novel variant at residue P1546 (p.P1546S), previously implicated by a different substitution (p.P1546A) in a patient with a severe, early-onset multisystem disorder, including cataplexy, narcolepsy, sensory neuropathy, hearing loss, myoclonus, immunodeficiency, and optic atrophy [24]. Although the p.P1546S variant segregated with disease in affected family members, incomplete segregation data limit definitive interpretation. Accordingly, under ACMG/AMP criteria, this variant remains classified as a variant of uncertain significance (VUS), emphasizing the need for additional functional studies or independent case reports.

Given the central role of DNMT1 in maintenance methylation, its dysfunction is expected to perturb DNA methylation homeostasis. Previous WGBS studies demonstrated global hypomethylation in blood-derived DNA from patients carrying the p.Y511C variant [6,9]. Consistent with these findings, nanopore-based methylation profiling revealed marked global hypomethylation in patients carrying the p.Y511H variant, supporting the quantitative reliability of nanopore sequencing. Importantly, nanopore long-read sequencing enables direct detection of cytosine methylation and simultaneous profiling across extended genomic regions, including repetitive and structurally complex loci, which are challenging to assess using short-read approaches.

In contrast, patients carrying other RFTS-domain variants (p.Y540C and p.H569R) exhibited global 5mC levels comparable to, or slightly higher than, those of controls. These findings indicate that global hypomethylation is not a universal hallmark of *DNMT1*-related disorders but rather a variant-specific outcome. This observation extends previous reports focused on p.Y511C by demonstrating heterogeneity in epigenomic states, even among variants affecting the same RFTS domain [6,9].

Notably, reduced enzymatic activity did not uniformly translate into global hypomethylation. The p.Y540C variant showed increased global 5mC levels despite reduced DNMT1 expression and activity in cellular assays. While counterintuitive, DNMT1 functions within a complex epigenetic network, and compensatory mechanisms involving other methyltransferases, chromatin context, or cellular selection may influence the net methylation outcome. In addition, peripheral blood-derived methylation profiles likely reflect cell population-level effects rather than direct enzymatic consequences. Accordingly, the observed methylation changes should be interpreted as associative rather than mechanistic.

CpG islands are enriched at gene promoters and regulatory elements and are typically unmethylated in a cell type-specific manner when associated with active transcription. Aberrant CpG island methylation has been implicated in neurodevelopmental and neurodegenerative disorders [25]. In our study, CpG island analysis revealed both hyper- and hypomethylated regions, in line with previous reports [6,9], indicating a complex and mosaic methylation landscape rather than uniform loss of methylation. Patients carrying the p.Y511H variant exhibited the lowest overall CpG island methylation, consistent with their global hypomethylation profile. Unsupervised PCA demonstrated that most *DNMT1* variant carriers exhibited methylation profiles distinct from controls, although substantial intra- and inter-individual variability was observed. In contrast, the p.A1334V variant clustered closely with controls, further supporting its limited epigenomic impact.

CpG island-level differential methylation analysis comparing controls and p.Y511H carriers identified a single significantly hypomethylated CpG island located within the *SLC6A18* locus. No statistically significant differences were detected at the other CpG islands annotated to this gene, suggesting that the observed change is region-specific rather than gene-wide. *SLC6A18* encodes a member of the solute carrier family involved in amino acid transport [26] and has not been previously associated with *DNMT1*-related disorders or established neurological phenotypes. Although CpG island hypomethylation is often associated with altered transcriptional regulation, the functional consequence of this isolated epigenetic change remains uncertain. Notably, this region does not overlap with previously reported *DNMT1*-specific episignature CpG sites [27], arguing against a canonical *DNMT1* methylation signature at this locus. Given the limited sample size and the modest effect size observed, this finding should be interpreted cautiously. Further studies incorporating transcript-level validation, expanded cohorts, and disease-relevant cellular models will be required to determine whether altered methylation at the *SLC6A18* locus represents a biologically meaningful consequence of the p.Y511H variant or a context-dependent epigenetic alteration.

5hmC is generated by TET enzymes and plays a critical role in active DNA demethylation, chromatin remodeling, and neuronal function. Perturbations in the balance between 5mC and 5hmC can disrupt gene regulation and have been implicated in neurodegenerative phenotypes [28]. In the present study, nanopore sequencing revealed variable 5hmC levels across patients but no significant difference compared with controls. These findings suggest that global 5hmC homeostasis may be largely preserved despite DNMT1 dysfunction, potentially through compensatory mechanisms, such as TET activity, chromatin remodeling, or histone modifications. This stability highlights the resilience of 5hmC landscapes and underscores the importance of assessing both 5mC and 5hmC to fully understand the epigenomic consequences of DNMT1 dysfunction.

Collectively, these findings establish nanopore sequencing as a powerful tool for comprehensive methylome profiling in *DNMT1*-related disorders. Although the mean genome coverage in this study was below 10×, previous work has shown that nanopore sequencing can provide reliable global methylation quantification concordant with bisulfite-based assays [12,13]. Peripheral blood DNA methylation episignatures, largely defined using bisulfite-based microarray platforms, have been described for several neurodevelopmental disorder-associated genes, including *DNMT1* [27], and have recently been validated using nanopore methylation analysis [29]. Because these episignatures are defined at the single CpG level, the relatively limited sequencing depth in our study did not allow comprehensive coverage of the signature CpG sites, limiting reliable discrimination between patients and controls. Moreover, our DMR-focused analytical approach does not fully exploit the long-range, single-molecule methylation context enabled by nanopore sequencing. Previous studies have shown that integrating episignature data with ACMG/AMP guidelines can improve variant interpretation, underscoring the clinical value of methylomic profiling [30]. Increasing sequencing depth in future studies will enhance data robustness and enable more effective integration of methylation signatures and genomic variant analysis.

GSEA revealed coordinated methylation changes at the pathway level despite the limited number of CpG island-level DMRs. Pathways enriched in the p.Y511H group were predominantly related to neurodevelopmental processes, including central nervous system neuron differentiation and cell fate commitment, consistent with the established role of DNMT1 in maintaining epigenetic programs during neural development. In contrast, pathways enriched in controls were associated with genome maintenance and mitochondrial function, such as DNA double-strand break repair and mitochondrial translation, suggesting relative disruption of these processes in p.Y511H carriers. Together, these findings support the notion that the p.Y511H variant leads to global hypomethylation accompanied by subtle but coordinated alterations in biologically relevant gene networks. However, given the modest effect sizes and limited cohort size, these pathway-level observations should be interpreted cautiously and warrant validation in larger cohorts and with transcriptomic analyses.

Our data did not reveal a simple relationship between clinical severity, in vitro enzymatic activity, and global methylation levels. For instance, P1 presented with a severe clinical phenotype despite showing global methylation levels comparable to controls, whereas P2 exhibited a typical phenotype with a methylation profile that clustered with controls. Likewise, variants associated with reduced enzymatic activity did not uniformly result in global hypomethylation, and epigenomic alterations varied among individuals carrying different *DNMT1* variants. Together, these observations indicate that *DNMT1*-related disorders are characterized by variant-specific and context-dependent molecular effects shaped by compensatory epigenetic mechanisms, tissue specificity, and cellular heterogeneity. Accordingly, the molecular alterations identified in this study should be interpreted as complementary features of *DNMT1* dysfunction rather than as direct predictors of clinical severity.

Several limitations should be acknowledged in this study. DNA methylation is highly cell type-specific, and leukocyte-derived profiles may not fully reflect neural epigenetic states. HEK293T cells, while useful for accessing cell-autonomous molecular effects, do not model post-mitotic neurons, and future studies using neuronal or patient-derived models will be essential. The small sample size, inclusion of disease controls rather than fully matched healthy individuals, and limited sequencing depth together constrained the scope and interpretability of the methylation analyses. Finally, integration with transcriptomic or proteomic data would strengthen mechanistic interpretation.

In conclusion, this study provides functional characterization of *DNMT1* variants across the RFTS and catalytic domains and presents the first genome-wide nanopore-based methylation analysis in *DNMT1*-related disorders. Our findings reveal variant-specific epigenetic signatures, refine genotype–phenotype correlations, and offer insights into domain-dependent and epigenetic mechanisms underlying *DNMT1*-related neurodegeneration.

## 4. Materials and Methods

### 4.1. Sample Collection

Eight patients from five unrelated pedigrees with a clinical diagnosis of HSAN and/or SCA were enrolled in this study. Comprehensive neurologic examinations were conducted by attending physicians at referring hospitals prior to genetic analysis. Peripheral blood samples were obtained, and genomic DNA was extracted using DNA extraction kits (QIAGEN, Hilden, Germany) according to the manufacturer’s instructions.

### 4.2. Gene Panel Sequencing and WES

For patients with HSAN (P1~3), targeted next-generation sequencing (NGS) was performed using a custom gene panel encompassing 18 HSAN-related genes: *ATL1*, *ATL3*, *CCT5*, *DNMT1*, *DST*, *ELP1*, *FLVCR1*, *KIF1A*, *NGF*, *NTRK1*, *PRNP*, *RETREG1*, *RNF170*, *SCN9A*, *SCN11A*, *SPTLC1*, *SPTLC2*, and *WNK1/HSN2*. Library preparation and sequencing were conducted on the Illumina MiSeq platform (Illumina, CA, USA), as previously described [31]. Sequence alignment and variant calling were performed using the CLC Genomics Workbench (QIAGEN, Hilden, Germany).

Genomic DNA from patients with SCA (P4, P5) was analyzed by WES using the Ion AmpliSeq library kit v2.0. Libraries were prepared with Ion PI Hi-Q chemistry on the Ion Chef Instrument, loaded onto Proton PI chips v3, and sequenced on the Ion Proton platform (Thermo Fisher Scientific, MA, USA). Data processing and variant calling were completed using Torrent Suite (v5.18) and Variant Caller software (v5.18) (Thermo Fisher Scientific, Waltham, MA, USA).

All sequencing reads were aligned to the human reference genome NCBI37/hg19. Detected variants from both gene panel sequencing and WES were analyzed using Ensembl Variant Effect Predictor (VEP) version 104 and in-house R scripts (R version 4.1.0). Population frequencies were cross-referenced with the Genome Aggregation Database (gnomAD; https://gnomad.broadinstitute.org; accessed on 1 September 2025) and the Japanese Multi Omics Reference Panel (jMorp; https://jmorp.megabank.tohoku.ac.jp/; accessed on 1 September 2025).

### 4.3. Site-Directed Mutagenesis and Transient Transfection

Full-length *DNMT1* cDNA (NM_001130823.3) was cloned into a pcDNA3.1 + C-6HIS vector (GenScript, Tokyo, Japan). Site-directed mutagenesis of identified variants was performed using the PrimSTAR Max mutagenesis kit (Takara bio, Otsu, Japan), and all constructs were verified by Sanger sequencing.

HEK293T cells (ATCC CRL-3216) were cultured in Dulbecco’s modified Eagle’s medium (DMEM) supplemented with 10% fetal bovine serum, 1% penicillin-streptomycin, and 2 mM L-glutamine at 37 °C with 5% CO_2_. Cells were transiently transfected with WT or variant *DNMT1* plasmids using Lipofectamine 3000 (Invitrogen, Waltham, MA, USA) and analyzed 24–48 h post-transfection.

### 4.4. Real-Time Quantitative PCR (RT-qPCR)

RNA was extracted with the Mag Extractor-RNA-Extraction Kit (TOYOBO, Osaka, Japan) and reverse-transcribed using the ReverTra Ace qPCR RT Kit (TOYOBO). RT-qPCR was performed using THUNDERBIRD SYBR qPCR Mix (TOYOBO) and gene-specific primers for *DNMT1* and *ACTB*. The cycling conditions were as follows: initial denaturation at 95 °C for 1 min, followed by 40 cycles of 95 °C for 15 s and 60 °C for 30 s. Reactions were performed on a StepOne Real-Time PCR system (Applied Biosystems) in technical triplicates with biological replicates, and relative expression levels were calculated using the 2^(-ΔΔCt) method with *ACTB* as the internal reference.

### 4.5. Western Blotting

Transfected cells were lysed in RIPA buffer (WAKO, Osaka, Japan) with 1% protease inhibitor cocktail. Protein concentration was measured using the Bradford Protein Assay Kit, and 20 μg of protein per sample was denatured in Laemmli Sample Buffer with 5% 2-mercaptoethanol, separated on 7.5% Mini-PROTEAN TGX Precast Protein Gels (Bio-Rad, Hercules, CA, USA), and transferred to PVDF membrane. Membranes were blocked with 4% Block Ace Powder (DS Pharma Biomedical Co., Ltd., Osaka, Japan) and probed with anti-DNMT1 (H-12, Santa Cruz, 1:500) and anti-GAPDH (016-25523, WAKO, 1:1500) mouse monoclonal antibodies, followed by HRP-conjugated secondary antibodies (anti-mouse; K4001; DAKO). Chemiluminescence was detected using Pierce ECL Western Blotting Substrate kit (Thermo Fisher Scientific) and imaged on a MultiImager II MultiBOX (Biotools, Gunma, Japan). Experiments were independently repeated three times. Densitometric analysis was performed using ImageJ (version 1.53k).

### 4.6. DNMT1 Activity Assay

His-tagged DNMT1 proteins were purified using the MagneHis Protein Purification System (Promega, WI, USA). Enzymatic activity was measured using the EpiQuik DNMT Activity/Inhibition ELISA Easy Kit (P-3139; Epigentek, Farmingdale, NY, USA). Equal amounts of purified DNMT1 protein (100 ng) were assayed in a microplate format, and absorbance was recorded using a Multiskan FC microplate reader (Thermo Fisher Scientific). For each independent experiment, DNMT1 activity values were normalized to the DNMT enzyme control provided with the kit. All assays were performed in duplicate or triplicate using three independent biological replicates.

### 4.7. Immunofluorescent Staining

Cells grown on poly-D-lysine-coated coverslips were fixed in 4% formaldehyde, permeabilized with 0.1% Triton X-100, and blocked with 5% normal goat serum + 2% BSA. Primary antibodies were anti-DNMT1 (mouse, H-12, 1:500) and anti-Cyclin B1 (rabbit, D5C10, Cell Signaling Technology, 1:500), followed by incubation with secondary antibodies, including Alexa Fluor 488 anti-rabbit IgG (A11034, Invitrogen) and Alexa Fluor 594 anti-mouse IgG (A11032, Invitrogen). Nuclei were stained with DAPI (1:1000; Thermo Fisher Scientific, Waltham, MA, USA). Images were acquired using a Zeiss LSM 900 confocal microscope (Carl Zeiss AG, Oberkochen, Germany).

### 4.8. Genome-Wide Methylation Profiling Using Nanopore Sequencing

Genomic DNA extracted from leukocytes of eight patients underwent whole-genome nanopore sequencing with 5mC/5hmC analysis. For comparison, five unrelated controls (three females and two males; age range, 24~68 years) were analyzed, including three patients with genetically undiagnosed peripheral neuropathy and two unaffected individuals.

DNA libraries were prepared using the Rapid Sequencing Kit (SQK-LSK108; Oxford Nanopore Technologies, ONT, Oxford, UK) and sequenced on the GridION platform with R9.4.1 flow cells (FLO-MIN106, ONT). Each sequencing run was performed for 72 h, with flow-cell washing using the Flow Cell Wash Kit (ONT). Basecalling and alignment to the GRCh38 reference genome were conducted using dorado (v0.5.0) and samtools, applying the super accurate model.

Genome-wide methylation calling and downstream analyses were performed using Modkit (v0.2.3). Global 5mC and 5hmC levels were quantified using the modkit summary function, with a confidence threshold of 0.8 applied for both read filtering and modification calling. Site-level CpG methylation was obtained using modkit pileup, and methylation values were subsequently aggregated at the CpG island level using the modkit dmr pair function. PCA was performed on CpG island-level methylation data using RStudio (v2023.9.1).

### 4.9. DMR Analysis and GSEA

CpG island-level methylation data from four patients carrying p.Y511H and five control individuals were analyzed in RStudio to identify differentially methylated CpG islands. Differential methylation results were visualized using a volcano plot generated with the ggplot2 package.

Genes associated with CpG islands were identified using BEDTools intersect by overlapping CpG island coordinates with UCSC HGNC gene annotations. For functional interpretation, GSEA was performed using fold change values derived from gene-level mean CpG island methylation between control and p.Y511H patient groups. GSEA was conducted in R using the clusterProfiler package, with enrichment analysis based on GO gene sets obtained via the msigdbr package. The top five significantly enriched pathways in both positive and negative directions were visualized using the dotplot function from the enrichplot package.

### 4.10. Statistical Analysis

Statistical methods used for each analysis are described in the corresponding Results sections and figure legends. All statistical analyses were performed using RStudio, and a two-sided *p* value < 0.05 was considered statistically significant unless otherwise specified. Data are presented as mean ± standard deviation (SD) unless stated otherwise.

## Figures and Tables

**Table 1:** Clinical features of eight patients from five pedigrees with *DNMT1* variants.

Patient	P1	P2	P3	P4	P5-1	P5-2	P5-3	P5-4
Clinical diagnosis	HSN	HSAN	HSN + SCA	SCA	SCA	SCA	SCA	SCA
*DNMT1* variant	H569R	A1334V	Y540C	P1546S	Y511H	Y511H	Y511H	Y511H
Gender	M	M	F	F	F	M	M	M
Age at evaluation	37	50	42	35	50	52	48	48
Age at onset	16	0	20	35	/	40	41	30
Family history	-	-	-	+	+	+	+	+
Motor function	-	+	-	-	/	-	-	-
Pain sensation	+	+	+	-	/	+	+	+
Vibration sensation	+	+	+	+	/	-	-	-
Autonomic function	-	+	-	-	/	-	-	-
Deep tendon reflex	Absent	Reduced	Absent	Reduced	/	Absent	Absent	Absent
Cerebellar ataxia	-	-	+	+	+	+	+	+
Cognitive impairment	+	+	+	-	/	+	+	+
Sleep disturbance	/	/	/	/	/	/	/	+
Hearing loss	+	+	+	-	/	+	+	+
Cataract	/	+	/	+	/	/	/	/
Cerebral atrophy (MRI)	+	+	-	+	/	+	+	+
Cerebellar atrophy (MRI)	+	-	+	-	/	+	+	+
SNAP	NE	/	NE	Reduced	/	NE	NE	NE

HSN (HSAN): hereditary sensory (and autonomic) neuropathy; SCA: spinocerebellar ataxia; M: male; F: female; +: abnormal or positive; -: normal or negative; /: no data; SNAP: sensory nerve action potential; NE: non-evokable.

**Table 2:** Global 5mC/5hmC proportions in control and patient samples.

Sample	Variant	5mC	5hmC	Unmethylated
C1	Control	0.698	0.058	0.244
C2	Control	0.675	0.066	0.258
C3	Control	0.674	0.057	0.269
C4	Control	0.677	0.074	0.248
C5	Control	0.692	0.069	0.238
P1	H569R	0.685	0.070	0.245
P2	A1334V	0.647	0.069	0.284
P3	Y540C	0.711	0.055	0.234
P4	P1546S	0.650	0.053	0.297
P5-1	Y511H	0.579	0.100	0.321
P5-2	Y511H	0.574	0.069	0.357
P5-3	Y511H	0.591	0.082	0.327
P5-4	Y511H	0.595	0.071	0.333

## Data Availability

The datasets presented in this article are not readily available because of ethical and privacy restrictions. Requests to access the datasets should be directed to the corresponding author.

## Supplementary Materials

The following supporting information can be downloaded at: https://www.mdpi.com/article/10.3390/ijms27031232/s1.
